# Progesterone Receptor Status and Ki-67 Index May Predict Early Relapse in Luminal B/HER2 Negative Breast Cancer Patients: A Retrospective Study

**DOI:** 10.1371/journal.pone.0095629

**Published:** 2014-08-29

**Authors:** Yu Zong, Li Zhu, Jiayi Wu, Xiaosong Chen, Ou Huang, Xiaochun Fei, Jianrong He, Weiguo Chen, Yafen Li, Kunwei Shen

**Affiliations:** 1 Comprehensive Breast Health Center, Shanghai Ruijin Hospital affiliated to Medical School of Shanghai Jiaotong University, Shanghai, China; 2 Pathology Department, Shanghai Ruijin Hospital affiliated to Medical School of Shanghai Jiaotong University, Shanghai, China; The Chinese University of Hong Kong, Hong Kong

## Abstract

**Purpose:**

Few studies has documented early relapse in luminal B/HER2-negative breast cancer. We examined prognostic factors for early relapse among these patients to improve treatment decision-making.

**Patients and Methods:**

A total 398 patients with luminal B/HER2-negative breast cancer were included. Kaplan-Meier curves were applied to estimate disease-free survival and Cox regression to identify prognostic factors.

**Results:**

Progesterone receptor (PR) negative expression was associated with higher tumor grade (p<.001) and higher Ki-67 index (p = .010). PR-negative patients received more chemotherapy than the PR-positive group (p = .009). After a median follow-up of 28 months, 17 patients (4.3%) had early relapses and 8 patients (2.0%) died of breast cancer. The 2-year disease-free survival was 97.7% in the PR-positive and 90.4% in the PR-negative groups (Log-rank p = .002). Also, patients with a high Ki-67 index (defined as >30%) had a reduced disease-free survival (DFS) when compared with low Ki-67 index group (≤30%) (98.0% vs 92.4%, respectively, Log-rank p = .013). In multivariate analysis, PR negativity was significantly associated with a reduced DFS (HR = 3.91, 95% CI 1.29–11.88, p = .016).

**Conclusion:**

In this study, PR negativity was a prognostic factor for early relapse in luminal B/HER2-negative breast cancer, while a high Ki-67 index suggested a higher risk of early relapse.

## Introduction

The most commonly diagnosed cancer among Chinese women in 2009 was of the breast, with an incidence of 42.55/10^5^, accounting for 16.8% of all new cancer cases among women [1]. Recent gene expression studies have confirmed that breast cancer is no longer a single disease with variable morphology, but with high molecular heterogeneity [2]. Breast cancer is now identified as at least 4 subtypes: Luminal A, Luminal B, HER2-enriched and basal-like by gene profiles [3], [4]. Since certain immunohistochemical (IHC) markers can provide prognosis and predictive treatment information and also have easy clinical accessibility [5], [6], these have been used in an attempt to determine intrinsic subtypes. A simplified IHC classification including estrogen receptor (ER), progesterone receptor (PR), HER2, and Ki-67 index is now considered a surrogate for establishing breast cancer subtype [7] and could be equivalent to intrinsic subtypes by gene profiling [8]–[10].

Luminal B breast cancer is classified as higher nuclear grade [2], lower expression of ER-related genes [11], [12], and higher expression of proliferative genes [13], [14] compared to luminal A, thus representing a heterogeneous disease. IHC classification defines Luminal B tumors as ER- and/or PR-positive, HER2-negative, and Ki-67 labeling index (Ki-67 index) ≥14%, or as ER- and/or PR-positive, HER2 over-expressed or amplified, any and Ki-67 index [7], although this is still a heterogeneous group. It has been reported that Luminal B had a 1.86 times greater risk of early relapse, but not late relapse, compared to luminal A [15]. Also, Luminal B cancers were relatively insensitive to endocrine therapy compared with Luminal A types [8] and to chemotherapy when compared with HER2-enriched and basal-like breast cancers [16]–[20]. In this study, we emphasized Luminal B/HER2-negative breast cancers since they lack the use of anti-HER2 treatment [15].

PR is an ER-regulated gene which mediates the effect of progesterone on the development of both the normal mammary gland and breast cancer [21]. The predictive value of PR status of the benefit of adjuvant tamoxifen in ER-positive breast cancer remains controversial [22]–[25]. It has recently been reported that PR loss is a prognosis factor in Luminal B breast cancers regardless of HER2 status [26].

Ki-67 index is a proliferation-related factor which associated with poor prognosis in breast cancer [27], [28]. Fourteen percent is adopted as the cutoff to identify Luminal A and B breast cancers in Europe [7] but not widely accepted in North America [29]. A promising Ki-67 index cut-off point for prognosis or treatment response prediction is lacking because of unreproducibility and analytical bias among different laboratories [30]. The clinical utility of Ki-67 index as a prognostic marker might be more apparent if it were considered within more narrowly defined tumor subgroups.

In order to get more information on Luminal B/HER2-negative breast cancer, we have attempted to identify prognostic factors for early relapse, as well as to define an appropriate Ki-67 index cutoff in our center which might predict early relapse. It is also important to at least add some data about breast cancer prognosis in Eastern countries and specifically in China, since etiologies and genetic background might lead to different form one area of the world to another.

## Patients and methods

### Patients

This retrospective study has been approved by the Ethical Committees of Shanghai Ruijin Hospital, whose results do not effect the treatment decision of any patient enrolled. All the patients provided their written informed consents to participant this study before the clinical and pathological data were collected. Using the Electronic Medical History Administration System V2.0 and database from Comprehensive Breast Health Center, Shanghai Ruijin Hospital, we collected information on consecutive breast cancer patients undergoing breast surgery between January 2009 and December 2011. Only Luminal B/HER2-negative breast cancer patients were included. Data on each patient's past medical history, current disease, surgical information, pathological results. and pathologic staging examinations (chest film/CT scan, upper abdominal ultrasonography, bone scan, etc.) were retrieved. Latest follow-up was at the end of June, 2013.

### Evaluation of ER, PR, HER2, Ki-67 index status

Tumors were classified histologically according to the World Health Organization Classification of Tumors [31]. Tumor staging was assessed according to American Joint Committee on Cancer (AJCC) Cancer Staging Handbook [32].

Histological grades and all biological features were evaluated based on the invasive components. Histological grade was evaluated according to Elston and Ellis scoring system [33]. We used a revised technique involves semiquantitative evaluation of three morphological features: the percentage of tubule formation, the degree of nuclear pleomorphism and an accurate mitotic count using a defined field area. A numerical scoring system was used and the overall grade is derived from a summation of individual scores for the three variables; three grades of differentiation were used. For tubule formation, 1 = >75% of the tumor area was composed of definite tubules, 2  = 10 to 75% of the area showed tubule formation, 3 = <10% of the area showed tubule formation. As to nuclear pleomorphism, 1 = Small, regular uniform cells, 2 = Moderate increase in size and variability, 3 = Marked variation. For mitotic counts, since we used Nikon Labophot microscope with ×40 objective, and a field area of 0.152 mm^2^, 1 = 0–5 mitoses per 10 fields, 2 = 6–10 mitoses per 10 fields, 3 = ≥11 mitoses per 10 fields. To obtain the overall tumor grade, the scores for each category were added together, giving a possible total of 3–9. Tumor grade was then allocated as 3–5 points: grade I, well-differentiated; 6–7 points: grade II, moderately differentiated; 8–9 points: grade III, poorly differentiated.

IHC staining of ER, PR, HER2 and Ki-67 was routinely carried out by using Ventana BenchMark XT system (Ventana Medical Systems, Tucson, AZ) in our hospital. All procedures were performed automatically in the BenchMark. IHC staining was performed on 4-µm slices of formalin-fixed paraffin-embedded (FFPE) tissue sections with primary antibodies against ER (SP1, 1∶100, Dako, Denmark), PR (PgR 636, 1∶100, Dako, Denmark), HER2 (4B5,Roche, Switzerland), Ki67(MIB-1, 1∶100, Dako, Denmark). The tissue sections were incubated with primary antibody of ER, PR and Ki67 for 32 minutes at 42°C and of HER2 for 16 minutes at 42°C. Sections were counterstained with hematoxylin.

A Fluorescence in situ hybridization (FISH) test for HER2 gene amplification was routinely ordered when HER2 was IHC 2+. FISH was performed using the PathVysion HER-2 DNA FISH Kit, (Vysis Inc, Downers Grove, IL) according to the manufacturer's instructions. Acid pretreatment and protease digestion were performed (Vysis paraffin pretreatment kit; Vysis Inc), followed by standard saline citrate (SSC) and formamide denaturation (72°C 5 minutes). After dehydration, the HER2/CEP 17 probe mixer was added. Slides were incubated in a moist chamber overnight at 37°C under a coverslip. On the following day, slides were washed in a stringency buffer (SSC, NP40), air-dried in the dark and incubated with 4,6 diamidino-2-phenylindole (DAPI) for nuclear identification.

The cutoff for ER positivity and PR positivity was 1% positive tumor cells with nuclear staining [34]. Positive for HER2 was either IHC HER2 3+_(defined as uniform intense membrane staining of >30% of invasive tumor cells) or FISH amplified (ratio of HER2 to CEP17 of >2.2) [35]. The Ki-67 index was expressed as the percentage of positively nuclear staining cells among at least 1000 invasive cells in the area scored. Staining intensity was not relevant [36].

### Follow-up and Statistical Analysis

Breast cancer relapse was defined as the first proven invasive local/contralateral breast, regional, or distant recurrence in any site [37]. First relapse would be biopsied if it was technically available but the biopsy was not compulsive. Recurrence and metastasis could be diagnosed base on clinical manifestation, physical examinations and radiographic images. Only invasive tumor recurrences were counted as a relapse and a recurrence with only in situ tumor was not counted as a relapse.

Follow-up and events were censored if they occurred beyond 2 years after diagnosis. The analysis thus focused exclusively on early relapse.

Chi-Square test was employed for categorized variables (Fisher's exact test when the Chi-square test was unavailable). Survival curves were plotted by Kaplan-Meier method. Log-rank test was used to determine the associations between individual variables and survival, logistic regression modeling to examine the association of tumor features with PR negativity and Cox proportional hazards regression analyses to identify significant prognostic factors in Luminal B/HER2-negative breast cancer. Statistical analyses were carried out in SPSS version 16.0(SPSS, Inc., Chicago, IL).

## Results

PR status was identified as negative in tumors from 101 of 398 patients (25.4%). One hundred and ninety-nine patients expressed PR with >1% to <20% of positive cells(50.0%), and all of which had weak to moderate staining intensity of PR. Conversely, the other 98 patients(24.6%) with staining percentage of PR-positive cells greater than 20% mostly had moderate to strong intensity. Patient and tumor characteristics of this cohort are summarized in Table 1 according to PR status.

**Table 1:** Association of patient and tumor characteristics with PR status.

Characteristics	Luminal B, HER2-, PR+	Luminal B, HER2-, PR−	*P*-value	Cases available (n)
Age (y/o), mean (±SD)	55.2(±12.8)	55.7(±12.4)	.195	398
Menstruation status			.100	398
Premenopausal	126(42.4%)	33(32.7%)		
Postmenopausal	171(57.6%)	68(67.3%)		
Histology			.835	398
IDC	273(91.9%)	92(91.1%)		
Non-IDC	24(8.1%)	9(8.9%)		
Tumor size (cm)			.280	393
≤2	188(63.7%)	56(57.1%)		
>2	107(36.3%)	42(42.9%)		
Positive ALN numbers			.784	392
0	177(60.6%)	64(64.0%)		
1∼3	71(24.3%)	21(21.0%)		
≥4	44(15.1%)	15(15.0%)		
Tumor grade			<.001	394
G2	190(64.8%)	39(38.6%)		
G3	76(25.9%)	51(50.5%)		
others	27(9.3%)	11 (10.9%)		
Ki-67 labeling index			.010	398
High	111(37.4%)	53(52.5%)		
Low	186(62.6%)	48(47.5%)		
Breast surgery			1.000	398
Mastectomy	248(83.5%)	84(83.2%)		
BCS	49 (16.5%)	17(16.8%)		
Chemotherapy			.009	348
Yes	137(52.7%)	61(69.3%)		
No	123(47.3%)	27(30.7%)		
Radiation Therapy			.615	363
Yes	88(32.3%)	34(37.8%)		
No	180(67.7%)	61(62.2%)		

Abbreviation: IDC = infiltrating ductal carcinoma, ALN = axillary lymph node, ER = Estrogen receptor, PR = progesterone receptor, BCS = breast conserving surgery.

The Ki-67 index was dichotomized to high (>30% immunoreactive cells) and low (≤30% immunoreactive cells) groups by using the median value of the Ki-67 index immunoreactivity as the cut-off point. One hundred and sixty-four of 398 patients (41.2%) were identified as Ki-67 index-high.

Treatment information was available in 348 patients. Generally, 198 patients received chemotherapy while another 150 patients did not. Chemotherapy regimens were given according to physicians' preference, including EC (Epirubicin 100 mg/m2 IV day 1, Cyclophosphamide 600 mg/m2 IV day 1, cycled every 21 days for 4 cycles), EC-T(Epirubicin 100 mg/m2 IV day 1, Cyclophosphamide 600 mg/m2 IV day 1, cycled every 21 days for 4 cycles followed by Docetaxel 100 mg/m2 IV on day 1, cycled every 21 days for 4 cycles), TEC (Docetaxel 75 mg/m2 IV day 1, Epirubicin 75 mg/m2 IV day 1, Cyclophosphamide 600 mg/m2 IV day 1, cycled every 21 days for 6 cycles), and TC (Docetaxel 75 mg/m2 IV day 1, Cyclophosphamide 600 mg/m2 IV day 1, cycled every 21 days for 4 cycles).

### Associations of PR status with patient and tumor characteristics

There was no correlation between PR and patient age (*p* = .195) or menstruation status (*p* = .100) (Table 1). PR negativity was associated with higher grade tumors (*p*<.001) and higher Ki-67 index (*p* = .010); PR-negative tumors received more chemotherapy (*p* = .009). In multivariable logistic regression modeling which included trial cohort and selected by tumor features, tumor grade was strongly associated with PR negativity (p<.001) (Table 2).

**Table 2:** Multivariable logistic regression model for PR negativity.

Characteristics	Estimate± SE	Odds Ratio (95%CI)	P-value
Grade III (vs. Grade II)	1.223±.264	3.396 (2.023–5.700)	<.001
High Ki-67 index (vs. low Ki-67 index)	.471±.251	1.601 (.979, 2.618)	.061

A significant correlation was identified between PR expression and Ki-67 index by Spearman Rank correlation. Luminal B/HER2- patients with PR negativity were more likely to have a Ki-67 index higher than 30% (r = −.138, p = .006).

### DFS and OS by PR status

At a median follow-up of 28 months (range 5.7–50.1 months), 17 patients (4.9%) had relapses, 7 in PR-positive group (2.4%) and 10 in PR-negative group (9.9%). Eight patients died due to breast cancer (2.0%). Among 17 patients with relapses, 6 patients had locoregional recurrences, 2 had contralateral breast cancers, and 9 had distant metastases. All the locoregional recurrence lesions and contralateral breast lesions were proven by using either fine needle aspiration or core needle biopsy. For the 9 patients with distant metastasis, the first metastatic lesion was biopsied in 4 patients (2 liver metastasis, 2 lung metastasis). The other 5 patients were diagnosed as metastatic breast caner based on clinical and radiographic findings.

**Figure 1:**
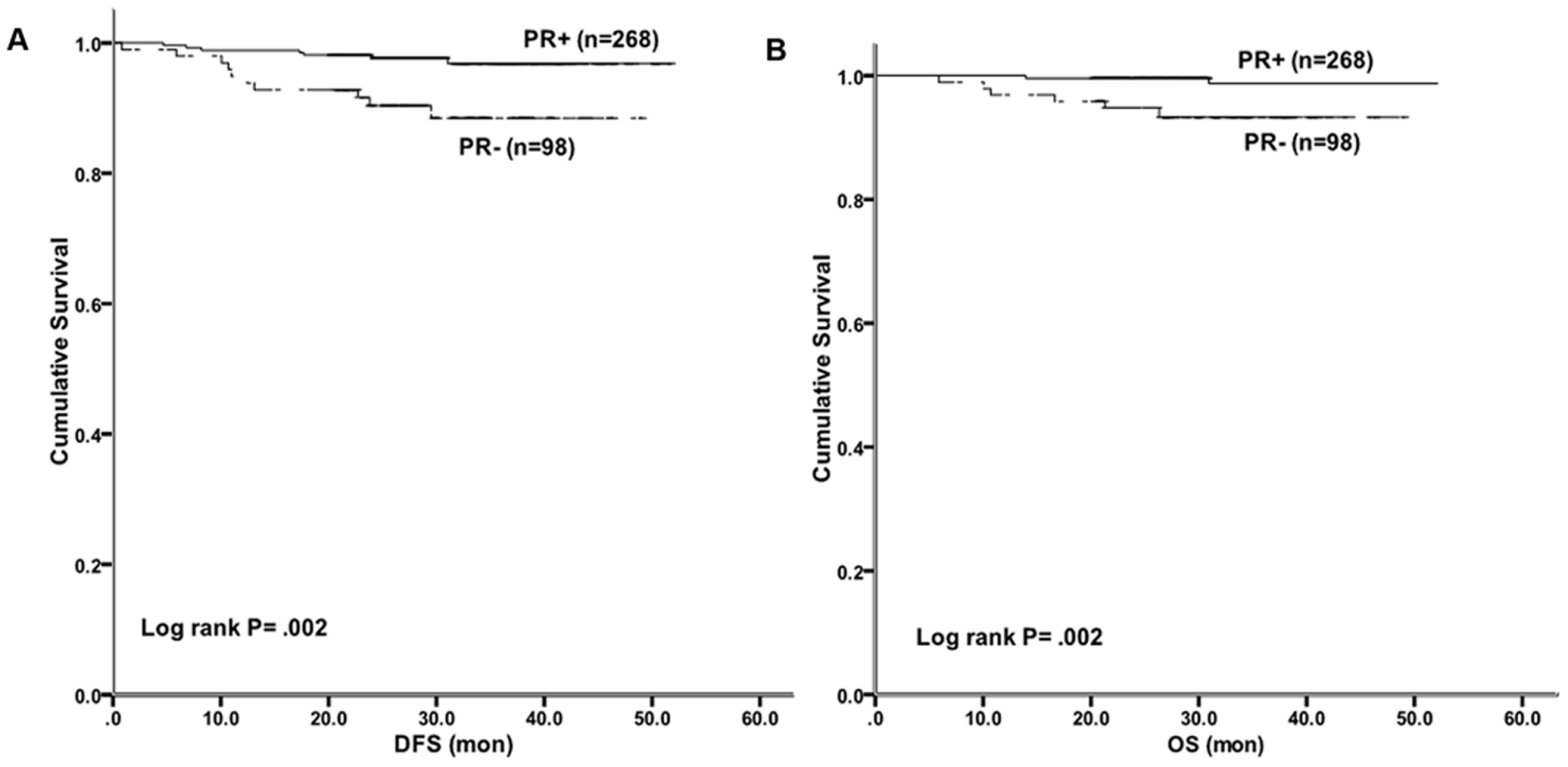
Disease-free survival and overall survival by PR status among Luminal B/HER2- patients. (A) The 2-year DFS was significantly better in the PR-positive group than in the PR-negative group (97.7% Vs 90.4%, Log-rank p = .002). (B) A poorer 2-year OS was also detected in PR-negative group (99.6% Vs 94.8%, Log-rank p = .002).

Information on 265 patients was available for Kaplan-Meier analysis. The 2-year DFS was 97.7% in the PR-positive group and 90.4% in the PR-negative group (Log-rank p = .002) (Figure 1A) in Kaplan-Meier analysis. A poorer 2-year OS was also detected in PR-negative group (99.6%%Vs 94.8%, respectively; Log-rank p = .002). (Figure 1B)

### DFS and OS by Ki-67 labeling index

**Figure 2:**
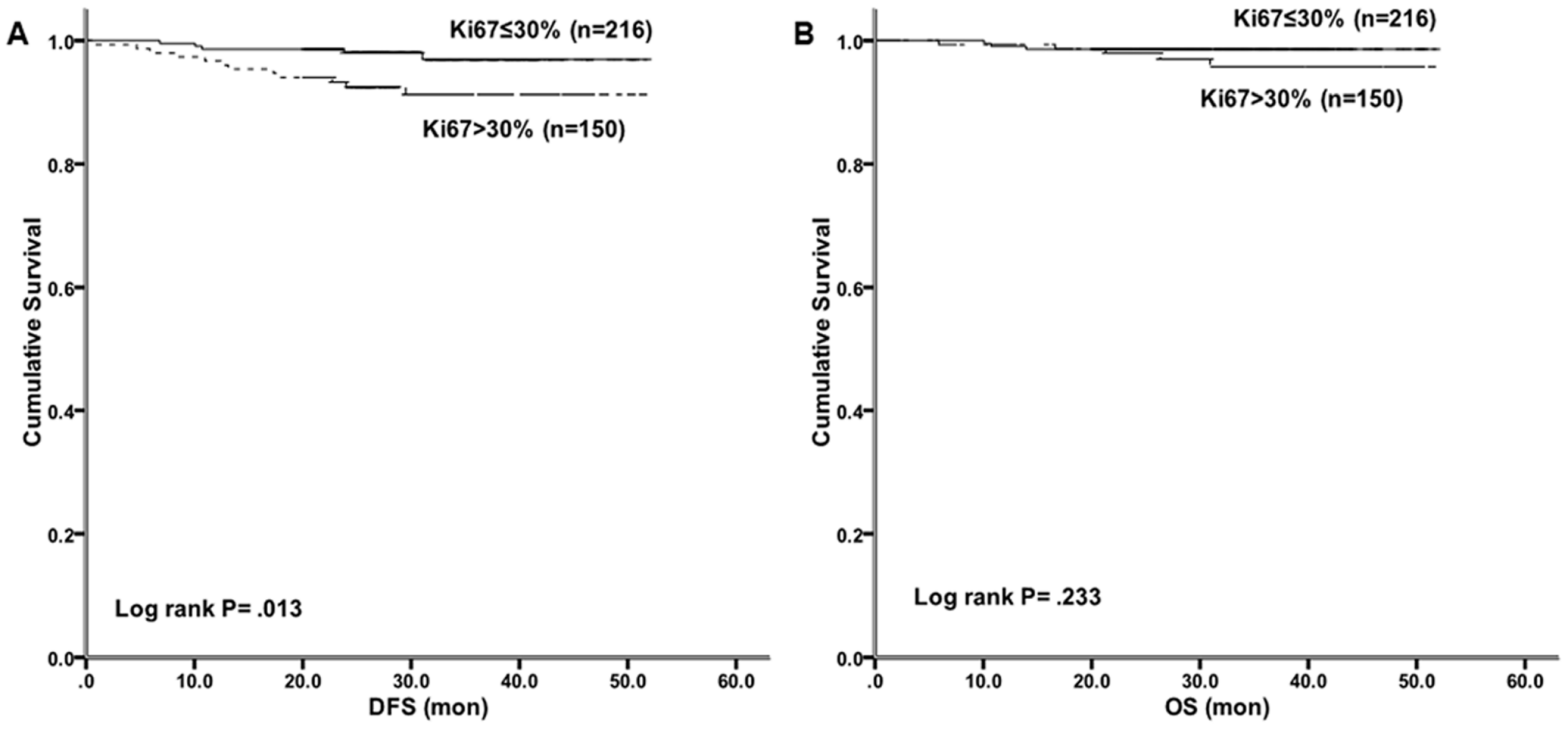
Disease-free survival and overall survival by high/low Ki-67 index among Luminal B/HER2- patients. (A) A significantly better 2-year DFS was detected in the high Ki-67 index group when compared to the low Ki-67 index group (98.0%Vs 92.4%, Log-rank p = .013). (B) No significant overall survival difference was detected between the high Ki-67 index group and the low Ki-67 index group (98.3% Vs 97.0%, Log-rank p = .233).

High Ki-67 index was significantly associated with poorer 2-year DFS (98.0%Vs 92.4% Log-rank p = .013; Figure 2A) in Kaplan-Meier analysis. However, No significant overall survival difference was detected between the high Ki-67 index group and the low Ki-67 index group (98.3% Vs 97.0%, Log-rank p = .233) (Figure 2B).

### Multivariate analysis of DFS

In univariate models, axillary lymph node status was the only insignificant covariate (Table 3). PR negativity (HR = 4.04, 95% CI 1.54–10.60; p = .005) and high Ki-67 index (HR = 3.45, 95% CI 1.22–9.80; p = .020) were both risk factors for early relapse in Luminal B/HER2-negative patients.

**Table 3:** Multivariate Cox proportional Hazards regression model results in Luminal B/HER2-negative tumors.

	Univariate		Multivariate	
	HR (95% CI)	P value	HR (95% CI)	P value
**PR**				
Positive	1.000		1.00	
Negative	4.04 (1.54–10.60)	.005	3.91 (1.29–11.88)	.016
**Ki-67**				
Low	1.000		1.00	
High	3.45 (1.22–9.80)	.020	2.54 (.79–8.20)	.119
**ALN**				
0	1.000		1.00	
1–3	.22 (.03–1.67)	.142	.23 (.03–1.79)	.160
≥4	1.93 (.54–6.83)	.309	2.34 (.63–8.66)	.204
**Chemotherapy**				
No	1.000		1.00	
Yes	9.40 (1.23–71.9)	.031	7.79 (0.99–60.77)	.050

Abbreviation: PR = progesterone receptor, Ki-67 = Ki-67 labeling index, ALN = axillary lymph node.

Since axillary lymph node status is a standard reported characteristic, it was included in the multivariate Cox proportional Hazards regression model. Results from the final multivariate Cox regression model are presented in Table 3. PR negativity was the only prognosis factor, resulting in an increased risk of relapse (HR = 4.031, 95% CI 1.293–12.574; p = .016).

### One representative case

**Figure 3:**
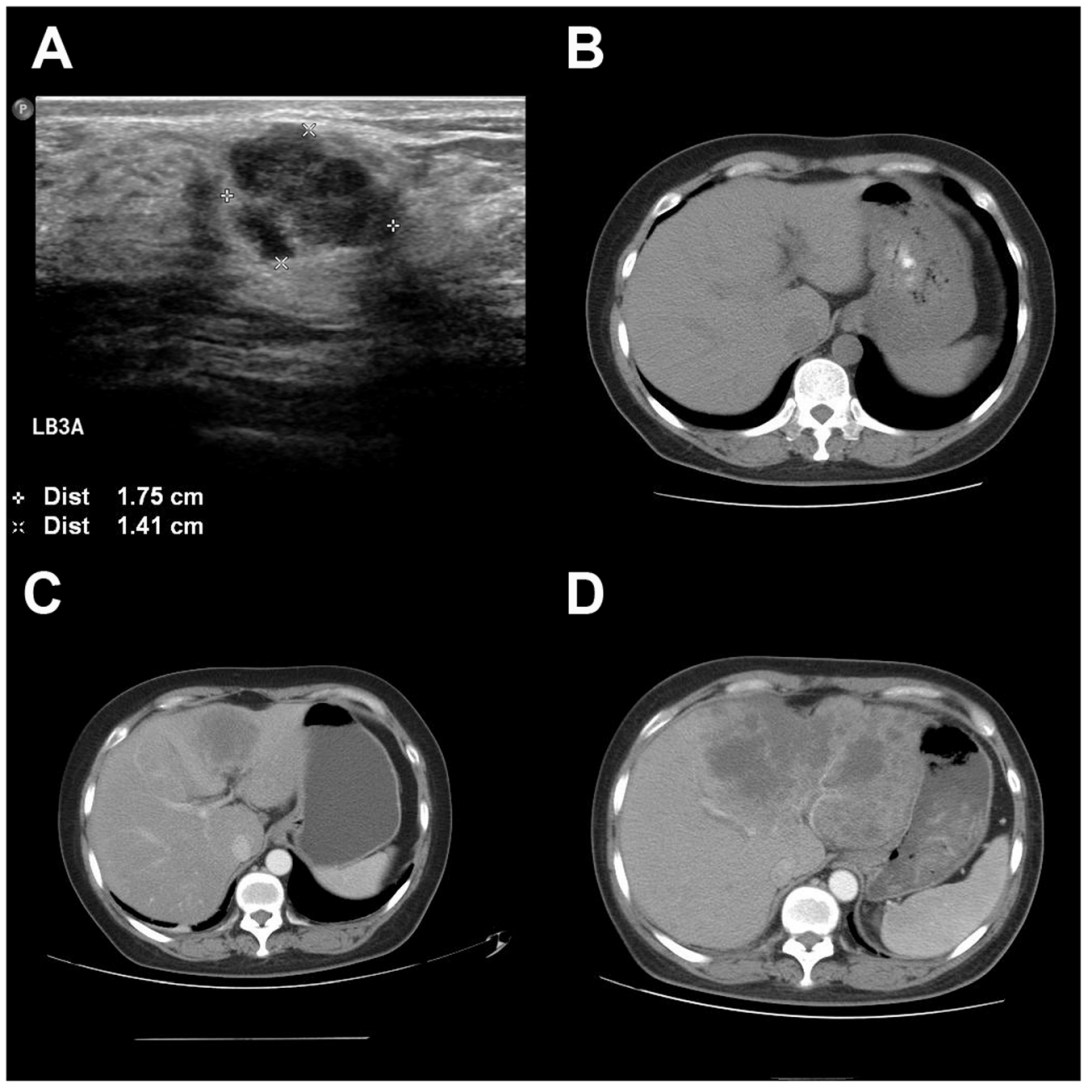
Representitive images of one patients with PR negative breast cancer. (A) Ultrasonography of the primary breast tumor before surgery (Dec, 2009). (B) No metastatic lesion showed on abdominal CT scan before surgery(Dec, 2009). (C) Liver metastasis confirmed by core needle biopsy 10 months after the surgery (Oct, 2010). (D) Last abdominal CT scan shortly before the patient died, which suggested no response to chemotherapy (Sept, 2011).

One representative patient was a 48 year-old female, diagnosed as infiltrating ductal carcinoma in Dec 2009. She received modified radical mastectomy. The pathology report showed IDC grade III, tumor size 1.5 cm, ALN 0/14, ER 10%+, PR−, HER2-, Ki-67 50%+. After 4 courses of TC (Docetaxel+Cyclophosphamide) chemotherapy, she received tamoxifen as adjuvant endocrine therapy. However, in Oct 2010, she received a abdominal CT scan due to abdominal discomfort, which suggested liver metastasis. Then, a CT-guided liver biopsy was done and the pathology report showed liver metastasis originated from breast cancer, ER-, PR−, HER2-, Ki-67 20%. She died after failing three lines of salvage chemotherapy in Sept, 2011. The images were showed in Fig. 3.

## Discussion

It is widely accepted that clinical factors combined with pathological characteristics and gene expression profiles provide useful prognostic information for the different breast cancer subtypes. However, Luminal B breast cancer, as one of the five intrinsic subtypes, still represents a heterogeneous disease, with distinct prognoses. Only one study has focused on Luminal B/HER2-negative breast cancer despite its tendency to early relapse [15]. Although the St. Gallen consensus recommends endocrine therapy±chemotherapy for the Luminal B/HER2-negative subtype [7], this subtype remains relative insensitivity to both treatments. In this study, we attempted to identify prognostic factors for early relapse in this subgroup.

PR is an estrogen-regulated gene, and its synthesis in normal breast and cancer cells requires estrogen and ER and PR expression represents a functional PR pathway [38]. PR-negative breast cancers are more frequent in postmenopausal patients [39], patients with a high BMI [40] and a high glucose intake [41]. This could be a result of loss of ovarian function in the elderly, causing insufficient levels of estrogen to transcribe PR [42].

In the 394 patients whose grade information was available, none had a grade I tumor, while in 38 patients, grade could not be established since they had non-infiltrating ductal carcinoma or micro-invasion. High tumor grade in present study is independently related to PR negativity after adjusting tumor size, axillary lymph node status, tumor histology. High Ki-67 index is also found significantly associated to PR status in Chi-square text and still has borderline meaning after multivariate logistic regression. Thus, we believe PR negativity in luminal B/HER2 negative breast cancer might suggest more aggressive tumor biology. Retrospective analysis showed that PR status did not affect decision making of surgery or radiation therapy. However, more chemotherapy was prescribed for those with PR negative luminal B/HER2 negative breast cancer.

Data are somewhat conflicting regarding the prognostic value of PR. According to an analysis by Bardou [22], PR negativity was an independent predictive marker for tamoxifen resistance and breast cancer relapse. Otherwise a meta-analysis by Early Breast Cancer Trialists' Collaborative Group (EGCTCG) found tamoxifen improves relapse-free survival in case of ER positive tumors, regardless of PR status [23]. More recently, a retrospective analysis by Cancello also identified PR negativity as high risk of relapse in luminal B breast cancer [26]. Our analysis showed that higher risk of early relapse was linked to PR negativity in luminal B/HER2 negative breast cancer in spite that chemotherapy and endocrine therapy was already employed. Several preclinical studies suggested that molecular crosstalk between ER and growth factor signaling pathways causes down-regulation of PR, activation of membrane initiated steroid signaling and tamoxifen resistance [43]–[45]. It is also observed that three times as many ER positive/PR negative tumors as ER positive/PR positive tumors expressed HER-1, which is associated with a higher likelihood of recurrence [46]. This crosstalk may explain the bad outcome of PR negative patients in our study.

Ki-67 index, a proliferation associated marker, has been widely accepted as a prognostic factor. Several studies suggested that higher values of Ki-67 index indicate a worse prognosis [27], [28]. Different methods were employed to identify Ki-67 index cut points for specific endpoints, such as median/mean value, ROC curves, or subpopulation treatment effect pattern plots (STEPP) analysis [8], [47], [48]. Instead of using any cut point as previously published, we chose median value of Ki-67 labeling index in our study population– 30% as cut-off point in case of reproducibility and analytical bias among different laboratories. Patients with high Ki-67 index (>30%) had more frequency having PR negative breast cancer according to logistic regression results and more risk of early relapse as univariate COX regression analysis presented but not multivariate models.

Despite of a median 28-month follow-up, PR might already show its prognostic value in luminal B/HER2 negative breast cancer and high Ki-67 index may also be an early relapse related factor. We also notice that the latest 2013 St Gallen Consensus employed high PR expression (>20%) to define a Luminal A subtype further [49]. Since all the patients in this study received surgery and adjuvant chemotherapy before 2013, the treatment strategies were given based on both tumor burden and intrinsic breast cancer subtypes, whose definition was from 2011 St. Gallen Consensus. Therefore, we did not use the new definition of Luminal B tumors in order to avoid bias in this retrospective study. Our results were also in line with that PR negativity was an unfavorable feature of breast cancer biology. However, previous data suggested that risk of death continues for an additional 10–15 years after diagnosis in HR positive/HER2 negative breast cancer [23], [50]. Therefore, longer follow-up period is warranted to identify whether PR status or Ki-67 index could still be a prognostic factor.

## Conclusion

PR status is a prognostic factor of early relapse in luminal B/HER2 negative breast cancer and high Ki-67 index may also suggest increased risk of early relapse. Therefore, PR status and Ki-67 index should be taken into account when discussing more aggressive treatment with specific luminal B/HER2 negative breast cancer patients.
